# Oral_voting_transfer: classification of oral microorganisms’ function proteins with voting transfer model

**DOI:** 10.3389/fmicb.2023.1277121

**Published:** 2024-02-07

**Authors:** Wenzheng Bao, Yujun Liu, Baitong Chen

**Affiliations:** ^1^School of Information Engineering, Xuzhou University of Technology, Xuzhou, China; ^2^The Affiliated Xuzhou Municipal Hospital of Xuzhou Medical University, Xuzhou, China; ^3^Department of Stomatology, Xuzhou First People’s Hospital, Xuzhou, China

**Keywords:** oral microorganisms proteins, voting transfer model, bioinformatics, machine learning, classification

## Abstract

**Introduction:**

The oral microbial group typically represents the human body’s highly complex microbial group ecosystem. Oral microorganisms take part in human diseases, including Oral cavity inflammation, mucosal disease, periodontal disease, tooth decay, and oral cancer. On the other hand, oral microbes can also cause endocrine disorders, digestive function, and nerve function disorders, such as diabetes, digestive system diseases, and Alzheimer’s disease. It was noted that the proteins of oral microbes play significant roles in these serious diseases. Having a good knowledge of oral microbes can be helpful in analyzing the procession of related diseases. Moreover, the high-dimensional features and imbalanced data lead to the complexity of oral microbial issues, which can hardly be solved with traditional experimental methods.

**Methods:**

To deal with these challenges, we proposed a novel method, which is oral_voting_transfer, to deal with such classification issues in the field of oral microorganisms. Such a method employed three features to classify the five oral microorganisms, including *Streptococcus mutans, Staphylococcus aureus*, abiotrophy adjacent, bifidobacterial, and *Capnocytophaga*. Firstly, we utilized the highly effective model, which successfully classifies the organelle’s proteins and transfers to deal with the oral microorganisms. And then, some classification methods can be treated as the local classifiers in this work. Finally, the results are voting from the transfer classifiers and the voting ones.

**Results and discussion:**

The proposed method achieved the well performances in the five oral microorganisms. The oral_voting_transfer is a standalone tool, and all its source codes are publicly available at https://github.com/baowz12345/voting_transfer.

## 1 Introduction

Microorganisms are a prominent type of biological group that are small and closely related to humans, including bacteria, viruses, fungi, small native creatures, and microalga (Xia et al., 2010; Ngounou Wetie et al., 2014). The efforts of biology have a history of more than a century. At the end of the 19th and early 20th century, Pasteur, Mechnikov, and Corch founded microbiology and micro-ecology (Zou et al., 2016). Since the discovery of Microorganisms, scholars have been committed to constructing and researching microbiological disciplines (Shen et al., 2007). The concept of Microbiota has become clearer with the in-depth study of microorganisms (Zhang et al., 2012). Microbial groups refer to the general name of all microorganisms in a specific time and specific growth environments, and the sum of the gene sequence contained in the microbial group contained in a specific time and specific growth environment is the microbiome group (Brohee and van Helden, 2006). EDERBERG first proposed the concept of the Human Microbiome in 2001, referring to all microorganisms living on the human body, including symbiotic and pathogenic bacteria, and the genetic sum of the corresponding microbial groups planted by the human body was called the human microbial group (Wu et al., 2009; Shen et al., 2019; Zhang and Zou, 2020).

The oral cavity is a complex micro-ecological environment. There are many types of oral microorganisms (De Las Rivas and Fontanillo, 2010; Wang et al., 2023). The oral microbial group is a typical representative of the human body’s highly complex microbial group ecosystem (Ngo et al., 1994; Whisstock and Lesk, 2003). Most of the oral microorganisms can be related to each other to form a complex community in the form of biofilm. It has strong physical resistance and biological resistance. When the host is balanced, the oral microorganisms can prevent the invasion of exogenous pathogenic bacteria. When changes in environmental changes or other oral conditions occur, such as personal oral hygiene care, They may become pathogenic microorganisms, which induce a variety of chronic infectious diseases of the oral cavity, including dental dentalism, pulp root disease, periodontal disease, wisdom tooth crown weekly Inflammation, mandibular osteomyelitis, etc., seriously endanger oral health (Godzik et al., 1995; Whisstock and Lesk, 2003; Peng et al., 2017; Awais et al., 2019). Studies have shown that the changes in oral microbial groups and the imbalance of oral micro-ecology are closely related to the occurrence and development of oral diseases such as oral cancer and diabetes (Chatterjee et al., 2011; You et al., 2013; Meng et al., 2023).

The protein function site prediction methods have been developed utilizing artificial intelligence techniques based on available data (Sato et al., 1994; Wei et al., 2017). It was noted that the traditional machine learning ones extract effective features manually to represent function site information, such as Shannon entropy, relative entropy, information gain, protein disordered property, and the average cumulative hydrophobicity (Schwikowski et al., 2000). With the development of artificial intelligence, deep-learning approaches with convolutional neural networks (CNNs) have been proposed to process raw sequence data without the need for manual feature extraction (Wenya et al., 2022). TAGPPI extracts multi-dimensional features by employing 1D convolution operation on protein sequences and graph learning method on contact maps constructed from AlphaFold (Song et al., 2022). Nguyen-Vo et al. (2022) proposed iPromoter-Seqvec, which is an efficient computational model to predict TATA and non-TATA promoters in human and mouse genomes using bidirectional long short-term memory neural networks in combination with sequence-embedded features extracted from input sequences. Robson et al. (2022) utilized ProSE to collect the custom heterogeneous systolic arrays and special functions. Several efforts have proposed various approaches for classification function sites in the field of bioinformatics and computational biology (Coates and Hall, 2003; Vazquez et al., 2003; Kim and Subramaniam, 2006; Free et al., 2009; Zhang et al., 2012). Wang et al. (2017) proposed a two-dimensional attention mechanism to classify the protein phosphorylation sites (Yang et al., 2020). In the same year, Wang et al. (2017) proposed a densely connected convolutional neuron network to identify the same PTM sites (Gao et al., 2023). Ahmed et al. employed a stacked long short-term memory recurrent network to deal with the function sites’ classification issue (You et al., 2017). Despite the immense potential of deep learning, a major challenge in developing classification methods based on this technology is the need for huge scale of computational resources. Therefore, the transfer learning has the ability to save the computing resources and improve computational reusability.

In this work, we proposed a novel method, which is oral_voting_transfer, to deal with such classification issues in the field of oral microorganisms. In detail, we utilized the highly effective model, which successfully classifies the organelles proteins and transfer to deal with five microorganisms, including streptococcus mutans, staphylococcus aureus, abiotrophia adjacens, bifidobacterial and capnocytophaga. It was noted that the transfer classifiers can hardly meet the need. On the other hand, some classification methods can be treated as the local classifiers in this work. And then, the final results are voting from the transfer classifiers and the voting ones. It was pointed out that the voting method employed three deep learning features, including TAGPPI, SeqVec, and ProSE. The detailed steps demonstrated in the following sections.

## 2 Materials and methods

### 2.1 Dataset

The identified datasets for this experiment was obtained from a protein-specific database website, which provides corresponding sequences and feature information for oral microorganisms (Guo et al., 2008; Torrey and Shavlik, 2010; Niu et al., 2020). In this experiment, we conducted qualitative analysis on the active sites and binding sites of five oral microorganisms. The details of each species are shown in Table 1.

**TABLE 1:** The information of the potential classify oral microorganisms.

Dataset	Active proteins	Non-active proteins	Binding proteins	Non-binding proteins
Streptococcus mutans	111	357	222	246
Staphylococcus aureus	203	860	496	567
Abiotrophia adjacens	283	3524	471	3336
Bifidobacterial	203	860	496	567
Capnocytophaga	283	4882	455	4710

The active proteins are the identified proteins with active sites in their sequences. And the non-active proteins are the proteins without any active sites. The terms, including binding and non-binding proteins, follow a similar procession in this work. Meanwhile, it is noted that there are three employed features in this work, including SeqVec, ProSE, and TAGPPI. Therefore, there are 30 identified datasets have been constructed.

### 2.2 Feature extraction based on deep learning datasets

#### 2.2.1 TAGPPI

This feature can be treated as a neural network algorithm called TAGPPI (TextCNN-based Approach for Gene-Protein Interaction Prediction), an end-to-end deep learning approach in computational proteomics. The feature utilizes a one-dimensional convolutional neural network (CNN) known as TextCNN, commonly used in natural language processing (NLP) tasks to capture the relationships between adjacent words in a sentence for classification purposes (Koike and Takagi, 2004).

The SCNN (Stacked Convolutional Neural Network) model in TAGPPI consists of six alternately stacked CNN and max-pooling layers (You et al., 2015b). The three combination layers are then stacked together. The architecture diagram of the sequence feature extraction and learning module.

The first step of this feature is to represent the entire amino acid sequence as a matrix, denoted as *X* ∈ R^L*M^, where M represents the number of selected feature dimensions (set to 1024), and L represents the maximum length of the given protein sequence (set to 1200) to ensure a fixed size of the output vector from the TextCNN module. For sequences shorter than 1200, zero padding is applied to the matrix before input. Therefore, the input matrix is denoted as *X* ∈ *R*^1200*1024^.

The SCNN model consists of a stack of six-layers CNN and max-pooling layers. In the first convolutional layer, 128 filters of length 3 are applied to the input matrix 1191×128, resulting in feature maps of 399×128. This is followed by a max-pooling layer with a pooling stride of 3, resulting in feature maps of the output matrix. These steps are repeated twice, resulting in a feature map of size 1×128. We have modified it to have six layers stacked, so the final obtained dataset is denoted as ℝ^*n*×768^, with a fixed dimension of 768 for all data.

With the above-mentioned processions, the input protein sequences are transformed into a matrix representation, which is then processed by the stacked CNN and max-pooling layers to extract sequential features. The output feature maps undergo further stacking and dimension reduction, resulting in a final dataset ℝ^*n*×768^ with a fixed dimensionality of 768.

#### 2.2.2 SeqVec

This feature can be regarded as a self-attentive deep learning algorithm based on ELMO for protein sequence generation. Since the ordered arrangement of amino acids in proteins follows a specific pattern, combining CharCNN and two LSTM layers is used to capture the surrounding words and context. The CharCNN converts all characters in a word into a vector space through an embedding layer and runs multiple CNNs to fix the vector dimensions (Dudoit et al., 2002; Lee et al., 2005). The first bidirectional LSTM takes the output of CharCNN as input, and the second bidirectional LSTM takes the output of the first LSTM as input.

The second layer, CharCNN, maps each amino acid to a fixed-length latent space of 1024 dimensions. The third layer, LSTM_1, introduces context-specific information by sequentially processing the protein sequence. The fourth layer, LSTM_2, directly operates on the output of LSTM_1 and attempts to predict all previous words in the protein sequence. During training, the forward and backward channels are independently optimized to reduce information leakage. Each layer ultimately generates a 1024-dimensional vector. The method add these vectors together to form a 1024-dimensional feature vector. Finally, the results of each protein sequence are combined to form the dataset ℝ*^n×1024^*, where the dimension of each data point is fixed at 1024. The variable n represents the number of identified datasets for the five oral microorganisms in this work.

#### 2.2.3 ProSE (protein sequence embeddings)

This feature is based on language modeling. Due to the influence of evolutionary pressures, protein functional properties and other characteristics are often maintained or enhanced in specific functions. These pressures are reflected in the distribution of amino acids in natural protein sequences (Bradford and Westhead, 2005; Díaz-Uriarte and de Andrés, 2006; Pashaei et al., 2016; Cui et al., 2012). The ProSE feature has been successfully utilized in several bioinformatics issues. Therefore, the ProSE feature effectively represents protein sequences as continuous vectors, combining the advantages of self-supervised learning on large-scale sequence corpora and structure-supervised learning on smaller sequence sets.

### 2.3 Transfer learning

**FIGURE 1:**
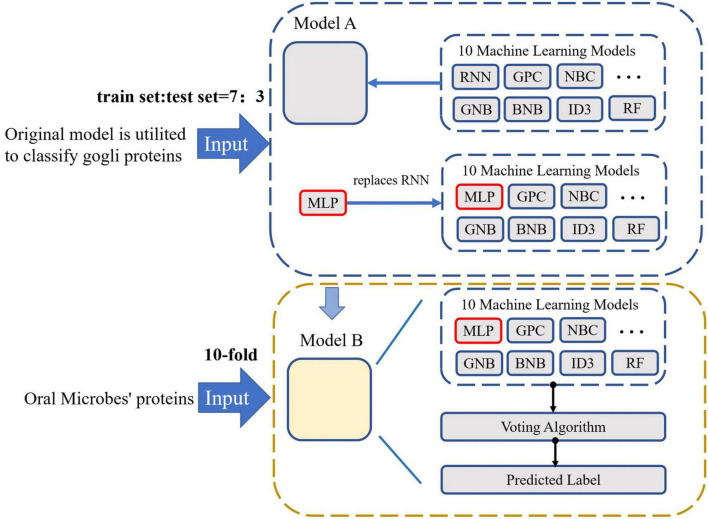
The flowchart of Oral_voting_transfer model

With the development of bioinformatics and computational biology, several artificial intelligence have been utilized in this field. Transfer learning is a novel machine learning approach. The field of data mining and machine learning has been successfully utilized in several areas (Chen and Liu, 2005; Li et al., 2012; Wang et al., 2018; Romero-Molina et al., 2019). First of all, the model utilized to classify the golgi proteins. Then, these ten classification models are transferred to classify the oral microorganisms’ proteins. Lastly, these models construct the voting transfer model to classify the Oral microorganisms’ proteins. The model structure is demonstrated in Figure 1.

### 2.4 Voting transfer learning model

In this work, we proposed oral_voting_transfer, which is the voting transfer learning method to deal with the classification of oral microbiology protein function sites. During this model, we employed several basic classifiers, including Gaussian Process Classification, Gaussian Naive Bayes, Bernoulli Naive Bayes, Decision Trees, Bagging meta-estimator, Random Forest, AdaBoost, Gradient Tree Boosting and Multilayer Perceptron (You et al., 2015a). In the following section, some typical methods can be shown.

#### 2.4.1 Random forest

The random forest algorithm was proposed by Arlot and Genuer (2016), and it is a very efficient general classification and regression technique (Sun et al., 2017). The method combines multiple probabilistic decision trees, aggregates average predictions, and works well in environments where the number of variables greatly exceeds the number of observations. Furthermore, it is general enough to be applied to large-scale problems, can be easily adapted to different specialized learning tasks, and provides multiple correlation metrics.

The algorithm works by growing M random trees as follows. Cases in the original dataset are randomly selected before building each tree. In each cell of each tree, splitting is performed by maximizing the basket criterion (see below) in m directions chosen uniformly and randomly from the original p directions. When the points of each cell are smaller than the node size, the construction of the tree ends. For any query point x∈X, each regression tree predicts the average of the *Y*_*i*_ for which the corresponding *X*_*i*_.

#### 2.4.2 AdaBoost

Boosting is an important method in blended learning. It provides new methods and ideas for designing learning algorithms. The idea behind it is to combine several weak learners that are slightly more accurate than random guessing to create arbitrarily accurate strong predictors (Zhang et al., 2019). This has important implications for situations where directly developing powerful learning algorithms is very difficult. As a meta-learning framework, Boosting can be applied to almost all popular machine learning algorithms to further improve prediction accuracy. Because of this, it is widely used and has had a major impact on the field of machine learning. Especially among all boosting algorithms, AdaBoost is the most successful representative, and is rated as one of the top ten algorithms in the field of data mining (Saha et al., 2014). Since AdaBoost was proposed, many excellent researchers have conducted research on various theoretical topics, laid a solid theoretical foundation for AdaBoost, and made contributions to the successful implementation of AdaBoost. The success of AdaBoost is not only because it is an effective learning algorithm, but also related to the following points.

### 2.5 Performances evaluation

Accuracy (Acc), sensitivity (Sn), specificity (Sp), and F1-score were used to evaluate the performance of the prediction system. were obtained using the formulae in Eqs 1–4, provided hereafter (Liu et al., 2018).


(1) $$S\,n=\frac{T\,P}{T\,P+F\,N}$$

(2) $$S\,p=\frac{T\,N}{T\,N+F\,P}$$

(3) $$\text{Acc}=\frac{T\,P+T\,N}{T\,P+T\,N+F\,P+F\,N}$$

(4) $$F\,1=\frac{2\,T\,P}{2\,T\,P+F\,N+F\,P}$$

(5) $$M\,C\,C=\frac{T\,P\times T\,N-F\,P\times F\,N}{\sqrt{\left(T\,P+F\,P\right)\,\left(T\,P+F\,N\right)\,\left(T\,N+F\,P\right)\,\left(T\,N+F\,N\right)}}$$

The performance of the classifier is evaluated using various metrics such as True Positives (TP), False Positives (FP), True Negatives (TN) and False Negatives (FN). Sensitivity (Sn) and specificity (Sp) measure the proportion of correct predictions for positive and negative samples, respectively. The F1 score reflects the strength of the model. The higher the score, the greater the resistance (Eq. 5). MCC is used to measure the strength of the linear relationship between two variables. The area under the ROC curve (AUC) was also used as an evaluation metric, with higher values indicating better model performance.

## 3 Results and discussions

### 3.1 The performances of transfer model

This is a machine learning-based voting ensemble strategy aimed at addressing the sub-classification task of Golgi apparatus proteins. This model can be employed as the transfer classification model. The Table 2 show performances the different ratios between positive and negative samples.

**TABLE 2:** The performances of different ratios.

Ratios	Sn	Sp	F1-score	MCC	ACC
0.1	100%	90.00%	0.9600	0.9045	97.29%
0.2	100%	95.00%	0.9800	0.9512	98.64%
0.3	100%	80.00%	0.9300	0.8165	94.59%
0.4	100%	85.00%	0.9500	0.8597	95.94%
0.5	100%	84.00%	0.9400	0.8510	95.65%

The results show that the proposed machine learning-based voting ensemble model for Golgi apparatus sub-classification achieves a stable accuracy of over 95% on the test set across different ratios of positive and negative samples in the training set. This indicates that the model has a high accuracy and generalizability. Overall, based on the results, the MVVM (Multi-Voting Machine Model) performs exceptionally well for Golgi apparatus sub-classification.

### 3.2 The performances of voting transfer learning

**FIGURE 2:**
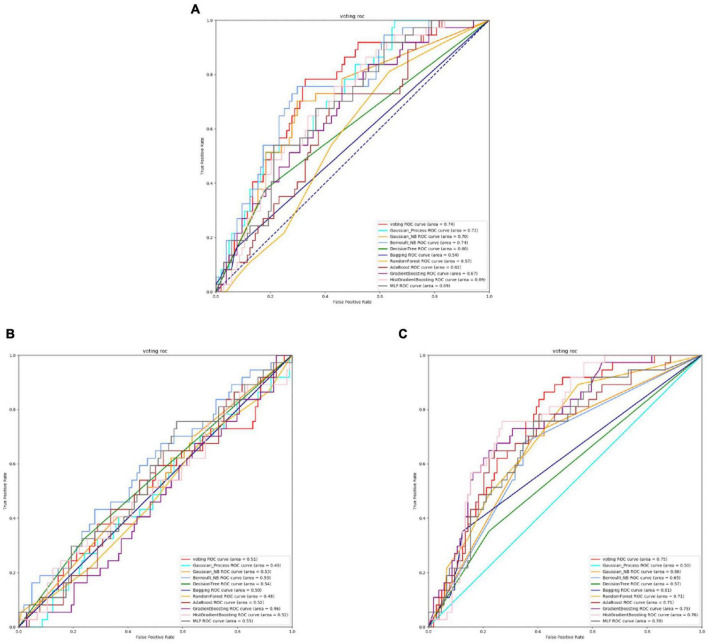
The ROC of Streptococcus mutans active sites. **(A)** Is the ROC curve of 10-fold cross validation with TAGPPI. **(B)** Is the ROC curve of 10-fold cross validation with SeqVec. **(C)** Is the ROC curve of 10-fold cross validation with ProSE.

**FIGURE 3:**
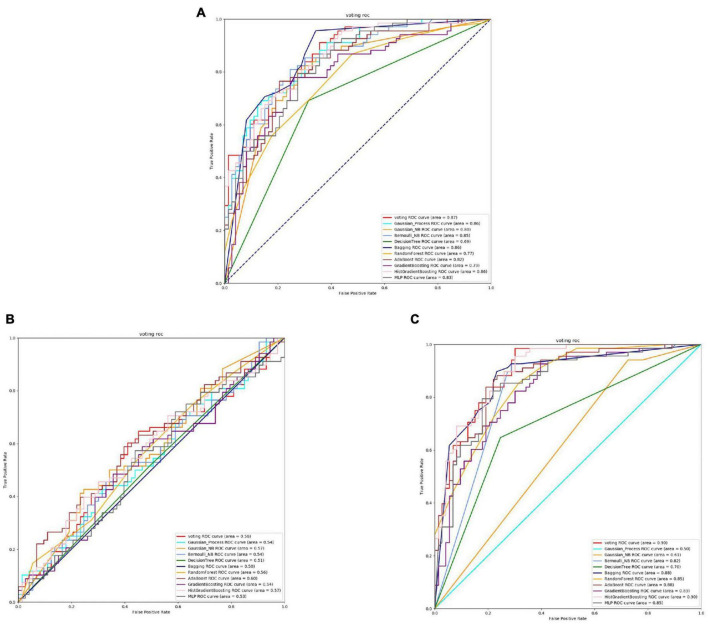
The ROC of Streptococcus mutans binding sites. **(A)** Is the ROC curve of 10-fold cross validation with TAGPPI. **(B)** Is the ROC curve of 10-fold cross validation with SeqVec. **(C)** Is the ROC curve of 10-fold cross validation with ProSE.

From Figures 2, 3 and Table 3, it can be observed that for Streptococcus mutans, the choice of feature extraction method has a limited impact on the accuracy of the active site prediction. However, the three different feature extraction methods still influence the model. The ProSE tends to provide better balance in terms of accuracy and positive sample coverage. SeqVec tends to overfit the positive samples and has a poorer balance. TAGPPI performs similarly to ProSE and has better coverage of positive samples but sacrifices some balance. Therefore, depending on the specific objective, ProSE is better when the balance is emphasized, while TAGPPI is preferable when emphasizing positive sample coverage.

**TABLE 3:** The performances of voting transfer learning model in Streptococcus mutans.

Site	Feature	ACC	Recall	F1	Sn	Sp	MCC
Active site	TAGPPI	73.05%	0.6000	0.6100	87.50%	32.43%	0.2388
	SeqVec	73.76%	0.5100	0.4500	99.04%	2.70%	0.0649
	ProSE	73.05%	0.5700	0.5800	90.38%	24.32%	0.1958
Binding site	TAGPPI	80.14%	0.8000	0.8000	80.82%	79.41%	0.6024
	SeqVec	56.03%	0.5600	0.5500	68.49%	42.65%	0.1153
	ProSE	75.18%	0.7500	0.7500	71.23%	79.41%	0.5081

Transfer learning offers notable advantages in terms of stability and coverage when it comes to identifying the active site and binding site of Streptococcus mutans. Moreover, the coverage of positive samples mostly exceeds 80%. According to the model accuracy for the binding site, it can be observed that the ProSE feature extraction method outperforms the other two methods. It has higher recall and F1 scores compared to the other methods. Therefore, ProSE is a better choice for identifying the binding site in Streptococcus mutans.

It is necessary to evaluate the specific feature selection for each microorganism using performance metrics and evaluate and optimize the overall transferred model. Below is the performance table for the Voting transfer learning models with three feature extraction methods for the active site.

According to the analysis of the table and ROC curves, it can be observed that the overall accuracy of the Gaussian Process and Bernoulli NB classification models is relatively low. This indicates that Bayesian classification models may not perform well for protein data where features are correlated. To further optimize the transfer learning, we can consider transforming the models or reducing the weight of the Bayesian model in the voting strategy.

By comparing the effect data of the classifier model in the voting and the effect of the transfer learning voting model, the accuracy and stability of the model have been optimized to a certain extent through the voting strategy. The table shows that TAGPPI, SeqVec, and ProSE have accuracy rates of 73.05, 73.76, and 73.05% respectively for the three features. The accuracy has been improved compared to the classification model in the voting process, indicating better stability and generalization than the trained model. On the other hand, the bagging algorithm and Decision Tree classification model consistently maintain high accuracy. We can consider increasing their weights appropriately to leverage their strong performance in the voting ensemble. The following is the scoring table for each model in the Voting transfer learning model of the Binding site in three feature extractions.

Based on the ROC curves, it can be observed that the green curve representing the Bernoulli NB classification model performs relatively poorly compared to the other models, and the difference is quite significant. The SeqVec feature extraction method, compared to the other two, shows overall lower training performance and is not suitable for feature extraction in binding sites.

In summary, the ProSE feature extraction method is more suitable for Streptococcus pneumoniae as it can extract sufficient features to ensure high accuracy in model training and prediction.

**FIGURE 4:**
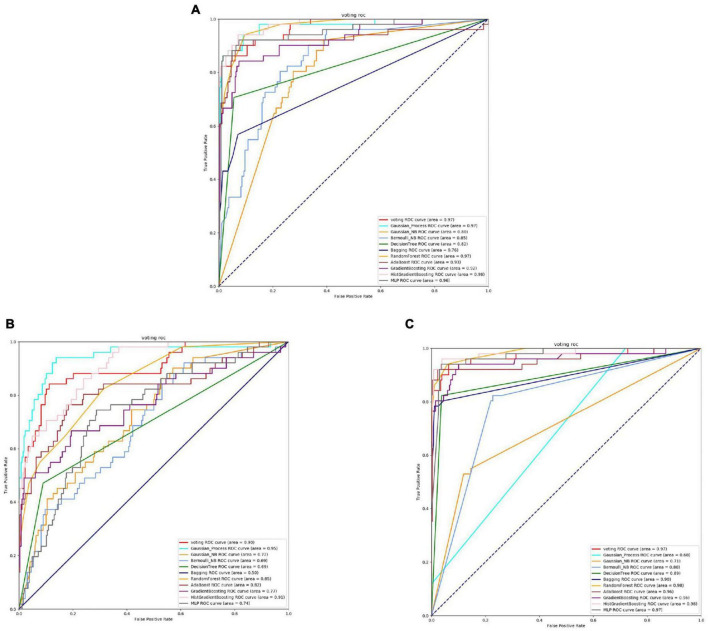
The ROC of Staphylococcus aureus active sites. **(A)** Is the ROC curve of 10-fold cross validation with TAGPPI. **(B)** Is the ROC curve of 10-fold cross validation with SeqVec. **(C)** Is the ROC curve of 10-fold cross validation with ProSE.

**FIGURE 5:**
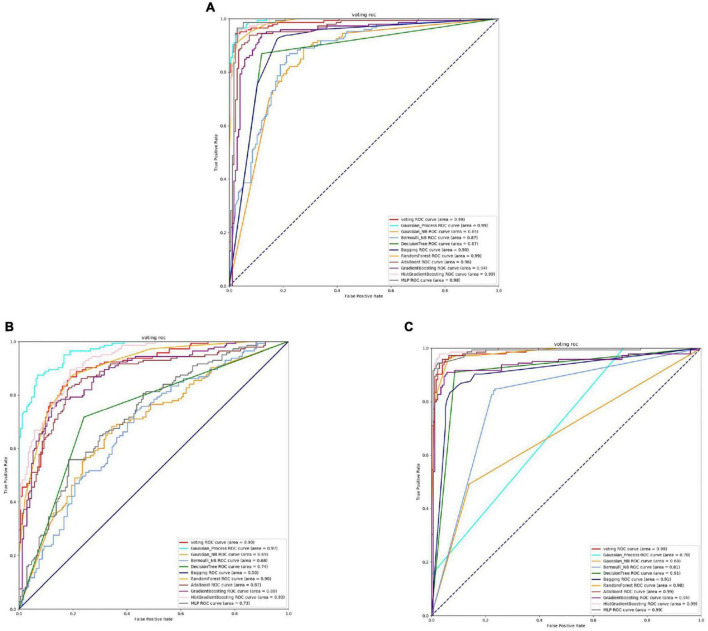
The ROC of Staphylococcus aureus binding sites. **(A)** Is the ROC curve of 10-fold cross validation with TAGPPI. **(B)** Is the ROC curve of 10-fold cross validation with SeqVec. **(C)** Is the ROC curve of 10-fold cross validation with ProSE.

From the Figures 4, 5 and Table 4, it can be seen that for Staphylococcus aureus, overall, the three feature extraction methods show excellent training results. However, SeqVec has the lowest accuracy, indicating that it is not suitable for Staphylococcus aureus. ProSE and TAGPPI, feature extraction methods, perform equally well, with accuracy above 90%, and exhibit good balance and coverage. Both methods can be applied to feature extraction in Staphylococcus aureus.

**TABLE 4:** The performances of voting transfer learning model in Staphylococcus aureus.

Site	Feature	ACC	Recall	F1	Sn	Sp	MCC
Active site	TAGPPI	94.98%	0.8700	0.9000	98.51%	76.47%	0.7687
	SeqVec	88.71%	0.6500	0.7000	100.00%	29.41%	0.4152
	ProSE	95.30%	0.8700	0.9000	99.25%	74.51%	0.7613
Binding site	TAGPPI	95.61%	0.9600	0.9600	95.98%	95.17%	0.9115
	SeqVec	82.76%	0.8200	0.8300	86.78%	77.93%	0.6496
	ProSE	93.42%	0.9400	0.9300	91.38%	95.86%	0.8733

The following is the score table of each model in the Voting transfer learning model of the active site in three feature extractions.

It can be observed that Gaussian Process and Neighbors have lower performance, while the models trained on the datasets extracted by TAGPPI consistently achieve high accuracy. This indicates that TAGPPI feature extraction is superior. Among the ProSE models, three of them show lower performance, and the ROC curve of SeqVec is more scattered. This suggests that the training of the models using these feature extraction methods is mostly based on weak learners. However, after applying the voting strategy, the accuracy is significantly improved, which validates the effectiveness of the transfer learning models. Through the evaluation indicators of each model, it can be observed that the recognition accuracy of Staphylococcus aureus’s active sites is relatively high. However, the indicators for model generalization and stability are inconsistent. After applying Voting Transfer Learning, the final model not only maintains an accuracy rate of more than 88% but also improves its stability. The following is the scoring table for each model in the Voting transfer learning model of the Binding site in three feature extractions.

The table data reveals that the accuracy of the model has improved after applying Voting Transfer Learning. Both the SN and SP index values are above 90%, indicating enhanced stability of the model through transfer learning.

The models trained on datasets extracted by TAGPPI consistently maintain high accuracy, indicating that TAGPPI feature extraction is superior. Among the ProSE models, three of them show lower performance, and the ROC curve of SeqVec is more scattered. This suggests that the training of these feature extraction models mostly relies on weak learners. However, after applying the voting strategy, the accuracy is significantly improved. This demonstrates that the transfer learning model is appropriate and effective.

**FIGURE 6:**
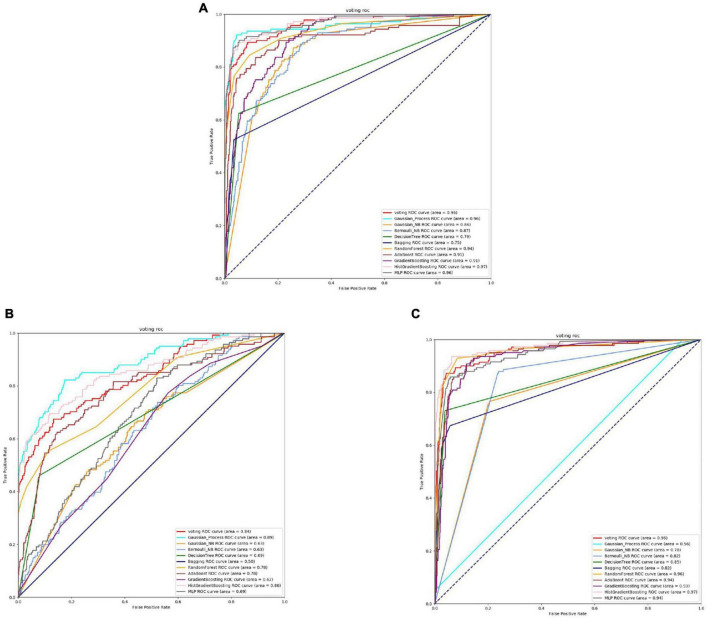
The ROC of Abiotrophia adjacens active sites. **(A)** Is the ROC curve of 10-fold cross validation with TAGPPI. **(B)** Is the ROC curve of 10-fold cross validation with SeqVec. **(C)** Is the ROC curve of 10-fold cross validation with ProSE.

**FIGURE 7:**
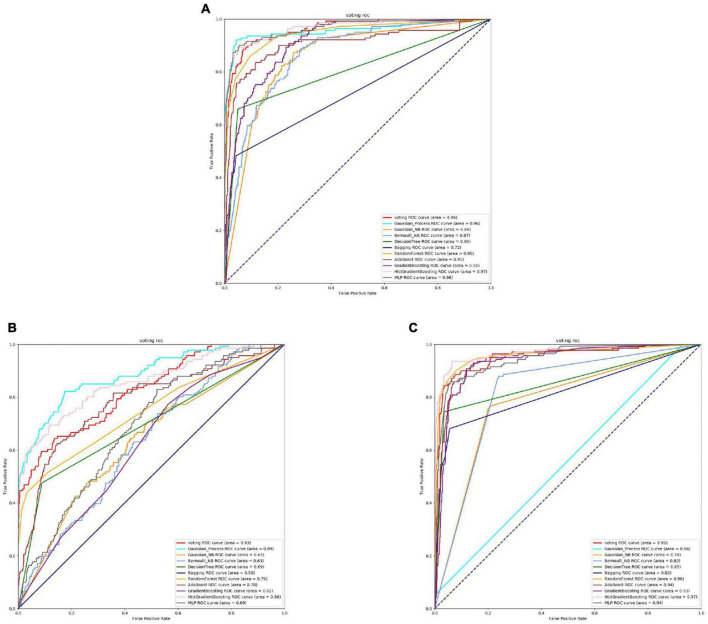
The ROC of Abiotrophia adiacens binding sites. **(A)** Is the ROC curve of 10-fold cross validation with TAGPPI. **(B)** Is the ROC curve of 10-fold cross validation with SeqVec. **(C)** Is the ROC curve of 10-fold cross validation with ProSE.

Based on the Figures 6, 7 and Table 5, we can observe that for Abiotrophia adjacens, overall, the three feature extraction methods yield good training results. However, there are some differences among them. SeqVec has the poorest balance, but it achieves 100% coverage of positive samples, indicating overfitting issues. Based on the data, we can conclude that TAGPPI is the optimal choice for both active site and binding site prediction in Abiotrophia adjacens, considering the model’s generalization, balance, and coverage.

**TABLE 5:** The performances of the voting transfer learning model in Abiotrophia adjacens.

Site	Feature	ACC	Recall	F1	Sn	Sp	MCC
Active site	TAGPPI	94.98%	0.8700	0.9000	98.51%	76.47%	0.7687
	SeqVec	88.71%	0.6500	0.7000	100.00%	29.41%	0.4152
	ProSE	95.30%	0.8700	0.9000	99.25%	74.51%	0.7613
Binding site	TAGPPI	95.61%	0.9600	0.9600	95.98%	95.17%	0.9115
	SeqVec	82.76%	0.8200	0.8300	86.78%	77.93%	0.6496
	ProSE	93.42%	0.9400	0.9300	91.38%	95.86%	0.8733

The following is the score table of each model in the Voting transfer learning model of the active site in three feature extractions.

It can be observed that the Radius Neighbors Classifier performs significantly worse than the other models, and Bernoulli Naive Bayes shows poor performance across all three feature extraction methods, indicating that it is a weak classifier. The ROC curve for SeqVec is more scattered, suggesting that SeqVec does not provide comprehensive feature extraction for the active site of Abiotrophia adjacens.

The following is the scoring table for each model in the Voting transfer learning model of the Binding site in three feature extractions.

Based on the ROC curves and the model performance tables, it can be observed that the active site and binding site exhibit high similarity. This suggests that Abiotrophia adjacens likely possesses both the active site and binding site in most cases, leading to the similarity in the dataset. This hypothesis needs to be verified, and a correlation analysis can be conducted between the active site and binding site of Abiotrophia adjacens to confirm this assumption. If the hypothesis is confirmed, the model can use the known sequence of one site to predict the presence of the other site with a high probability, thus improving the efficiency and accuracy of the model training.

**FIGURE 8:**
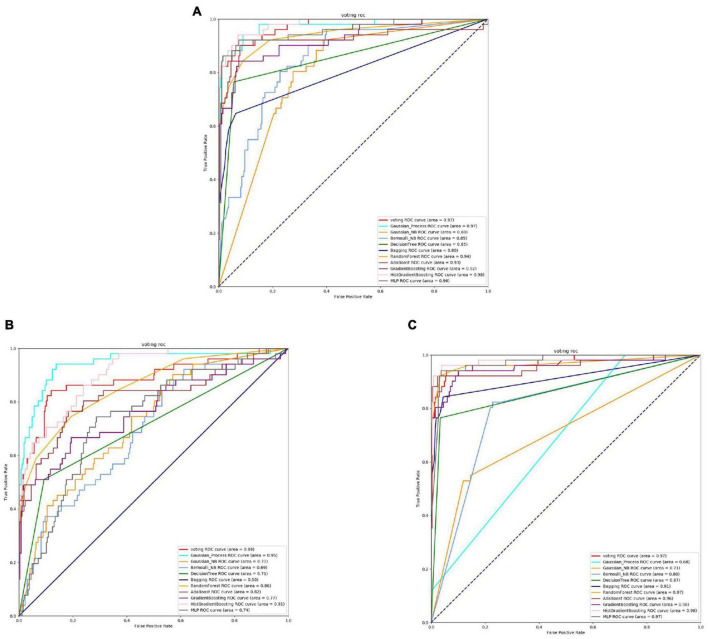
The ROC of Bifidobacterial active sites. **(A)** Is the ROC curve of 10-fold cross validation with TAGPPI. **(B)** Is the ROC curve of 10-fold cross validation with SeqVec. **(C)** Is the ROC curve of 10-fold cross validation with ProSE.

**FIGURE 9:**
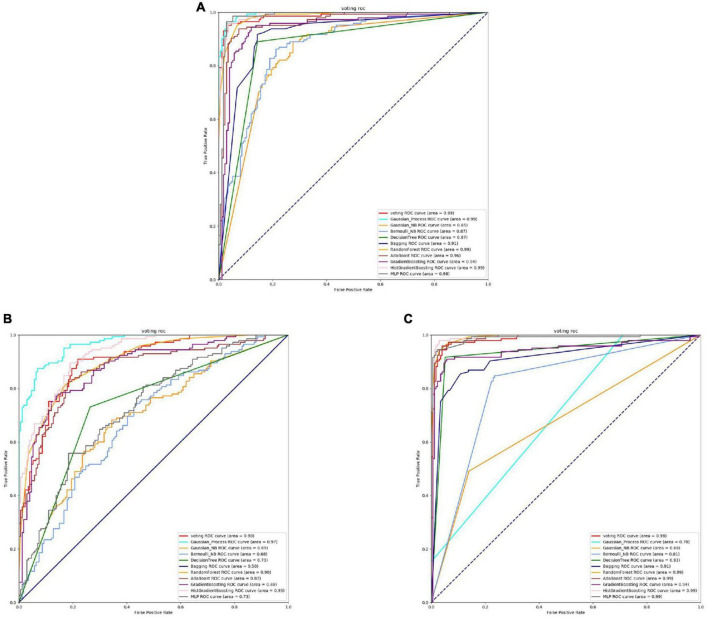
The ROC of Bifidobacterial binding sites. **(A)** Is the ROC curve of 10-fold cross validation with TAGPPI. **(B)** Is the ROC curve of 10-fold cross validation with SeqVec. **(C)** Is the ROC curve of 10-fold cross validation with ProSE.

Based on Figures 8, 9 and Table 6, it can be observed that for Bifidobacterium, overall, the three feature extraction methods yield good results in model training, but there are differences among them. SeqVec has the poorest balance and the lowest accuracy, sacrificing generalization to achieve a high positive sample coverage rate, which makes it relatively inferior compared to the other two methods.

**TABLE 6:** The performances of the voting transfer learning model in Bifidobacterial.

Site	Feature	ACC	Recall	F1	Sn	Sp	MCC
Active site	TAGPPI	95.30%	0.8800	0.9100	98.88%	76.47%	0.7732
	SeqVec	89.34%	0.6700	0.7200	100.00%	33.33%	0.4472
	ProSE	95.30%	0.8700	0.9000	99.25%	74.51%	0.7613
Binding site	TAGPPI	94.67%	0.9500	0.9500	95.98%	93.10%	0.8912
	SeqVec	81.19%	0.8000	0.8100	89.08%	71.72%	0.6174
	ProSE	93.42%	0.9400	0.9300	91.95%	95.17%	0.8717

According to the data, it can be concluded that both ProSE and TAGPPI exhibit good coverage and balance for the active site and binding site of Bifidobacterium. Both feature extraction methods are viable options, but considering model accuracy, ProSE is the optimal choice. However, if factors such as training cost and large-scale application are taken into account, TAGPPI, which has lower dimensionality, is more suitable.

The following is the score table of each model in the Voting transfer learning model of the active site in three feature extractions.

It can be observed that the Gaussian Process consistently performs poorly, and the ROC curve for the SeqVec feature extraction method is scattered. This clearly indicates that SeqVec does not provide comprehensive feature extraction for the active site of Bifidobacterium. This issue was also evident in the previous analysis of neighboring poor-nutrition bacteria, suggesting that SeqVec feature extraction method is not suitable for some microorganisms and yields subpar results. To improve experimental efficiency, one approach could involve identifying protein sequences with poor performance and dividing them into categories of weakly effective proteins.

The following is the scoring table for each model in the Voting transfer learning model of the Binding site in three feature extractions.

Based on the ROC curves and model evaluation scores, it can be observed that the active site and binding site exhibit high similarity, and Gaussian Process consistently performs poorly. The ROC curve for SeqVec feature extraction method is scattered.

In summary, for Bifidobacterium, using TAGPPI for feature extraction is the optimal choice. It yields high accuracy for both the transfer learning model and the training model. SeqVec feature extraction method is not suitable for some microorganisms and produces inferior results. To improve experimental efficiency, one approach could involve categorizing proteins with poor performance and identifying weakly effective protein sequences.

**FIGURE 10:**
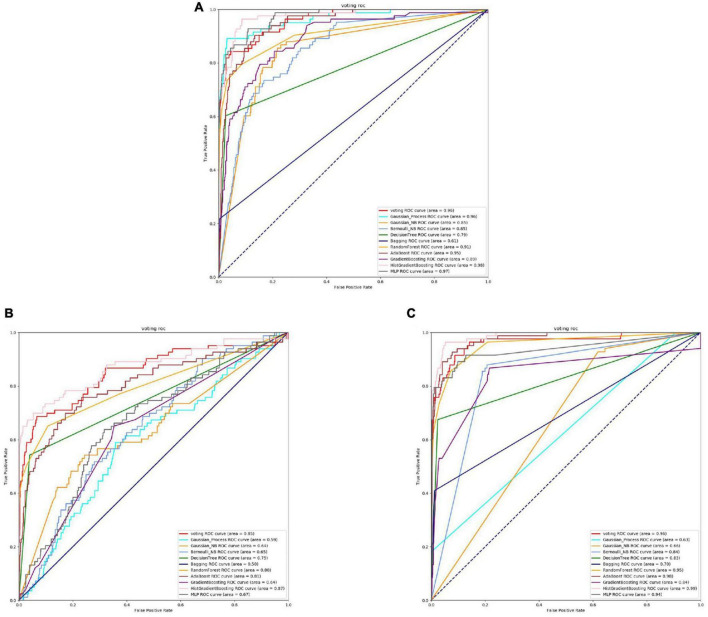
The ROC of Capnocytophaga active sites. **(A)** Is the ROC curve of 10-fold cross validation with TAGPPI. **(B)** Is the ROC curve of 10-fold cross validation with SeqVec. **(C)** Is the ROC curve of 10-fold cross validation with ProSE.

**FIGURE 11:**
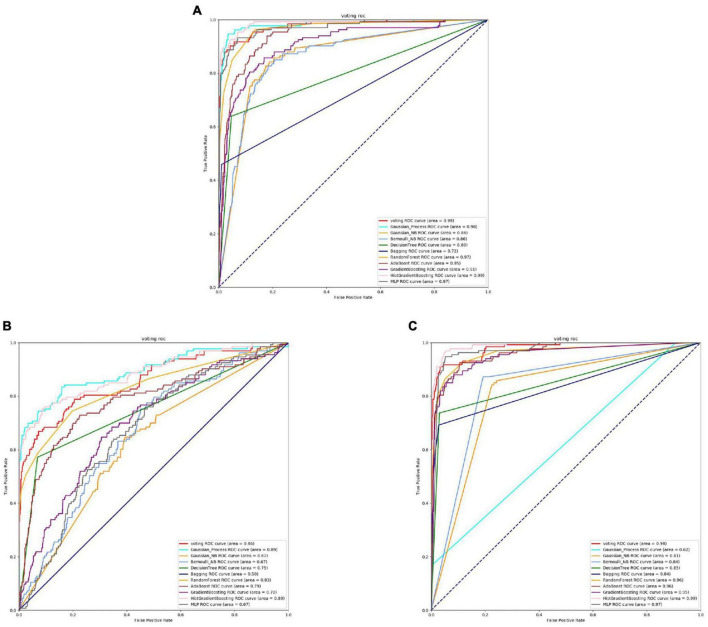
The ROC of Capnocytophaga binding sites. **(A)** Is the ROC curve of 10-fold cross validation with TAGPPI. **(B)** Is the ROC curve of 10-fold cross validation with SeqVec. **(C)** Is the ROC curve of 10-fold cross validation with ProSE.

The Figures 10, 11 and Table 7 shows that, overall, the three feature extraction methods perform well for Capnocytophaga. However, SeqVec exhibits relatively poorer performance and even has a case where sp = 0, indicating that this feature extraction method predominantly captures positive site-specific features while weakening the negative site-specific features. On the other hand, the other two feature extraction methods not only preserve positive site-specific features but also maintain a coverage rate of around 60% for negative site-specific features. Therefore, ProSE and TAGPPI are more suitable for Capnocytophaga.

**TABLE 7:** The performances of the voting transfer learning model in Capnocytophaga.

Site	Feature	ACC	recall	F1	Sn	Sp	MCC
Active site	TAGPPI	97.68%	0.8600	0.8800	99.05%	73.49%	0.7503
	SeqVec	94.65%	0.5000	0.4900	100.00%	0.00%	0.0000
	ProSE	97.35%	0.7800	0.8400	99.66%	56.63%	0.6236
Binding site	TAGPPI	97.55%	0.8800	0.9200	99.44%	77.44%	0.7881
	SeqVec	93.48%	0.6200	0.6800	99.93%	24.81%	0.3748
	ProSE	97.03%	0.8500	0.8900	99.58%	69.92%	0.7277

The following is the score table of each model in the Voting transfer learning model of the active site in three feature extractions.

It can be observed that the Decision Tree model performs relatively poorly, while both ProSE and TAGPPI show relatively higher performance compared to the previous cases, although they both exhibit weak classifiers. In this case, weak classifiers are present in both methods, but ProSE has a slightly higher number of weak classifiers. However, the final accuracy of the transfer learning models is comparable. SeqVec ROC curve is more scattered, indicating that the majority of the models trained using this feature extraction method are weak learners, resulting in significantly worse performance compared to the other two methods. However, after applying the voting strategy, the accuracy is only slightly lower than the other two methods, by around 5%. Overall, this demonstrates the excellent generalization ability of the models.

The following is the scoring table for each model in the Voting transfer learning model of the Binding site in three feature extractions.

The models trained using the TAGPPI feature extraction method consistently exhibit high accuracy, indicating that TAGPPI is a superior feature extraction method. Compared to the active site, it can be observed that there is a significant similarity in both the overall dataset and model results for the binding site. This suggests that the active site and binding site in the Capnocytophaga may have similar feature labels or exhibit some degree of overlap. The SeqVec feature extraction method still shows scattered ROC curves, indicating its ongoing issues with training models effectively.

Based on the above analysis, the following feature extraction methods are recommended for the discrimination of the five oral microorganisms: Streptococcus mutans: ProSE; Staphylococcus aureus: ProSE or TAGPPI; Abiotrophia adjacens: ProSE or TAGPPI; Bifidobacterial: TAGPPI; Capnocytophaga: TAGPPI. The five oral microorganisms will be judged as a whole.

According to Table 8, it can be observed that TAGPPI performs poorly for the Streptococcus mutans model. However, for the remaining four species, the models trained using TAGPPI achieve excellent results with an accuracy of around 95%. Upon observing the F1 scores, it is evident that most of them are above 0.85, indicating that the models trained on TAGPPI’s dataset are stable and comparable to ProSE. The recall values are also generally around or above 0.85. This suggests that TAGPPI is the most effective feature extraction method among the three for this particular transfer learning model.

**TABLE 8:** Voting transfer learning model with TAGPPI feature performances.

Sites	Microbe	ACC	Recall	F1	Sn	Sp	MCC
Active site	Streptococcus	73.05%	0.5700	0.5800	90.38%	24.32%	0.1958
	Staphylococcus	95.30%	0.8700	0.9000	99.25%	74.51%	0.7613
	Abiotrophia	94.66%	0.8300	0.8600	98.50%	67.38%	0.6932
	Bifidobacterial	95.30%	0.8700	0.9000	99.25%	74.51%	0.7613
	Capnocytophaga	97.35%	0.7800	0.8400	99.66%	56.63%	0.6236
Binding site	Streptococcus	75.18%	0.7500	0.7500	71.23%	79.41%	0.5081
	Staphylococcus	93.42%	0.9400	0.9300	91.38%	95.86%	0.8733
	Abiotrophia	94.58%	0.8300	0.8600	98.50%	66.67%	0.6875
	Bifidobacterial	93.42%	0.9400	0.9300	91.95%	95.17%	0.8717
	Capnocytophaga	97.03%	0.8500	0.8900	99.58%	69.92%	0.7277

According to the Supplementary Tables 2, 3, it can be observed that SeqVec performs poorly for the Streptococcus mutans model. For the remaining four species, the models achieve good results with accuracy above 80%, which is considered excellent. However, upon observing the F1 scores, it is found that most of them are below 0.5, indicating a lack of stability in the models. This is something that should be avoided in practical applications. Considering the recall values, only Staphylococcus aureus and Bifidobacterium show relatively good performance. It is also noted that SeqVec exhibits high coverage for positive instances, indicating an emphasis on extracting features related to positive class labels. This can be considered the optimal solution for specific requirements. Therefore, overall, SeqVec is not suitable for all oral microbiota and has its limitations.

## 4 Conclusion

In this work, we proposed a novel method, which is oral_voting_transfer, to deal with such classification issues in the field of oral microorganisms. In detail, we utilized the highly effective model, which successfully classifies the organelles proteins and transfer to deal with five microorganisms, including streptococcus mutans, staphylococcus aureus, abiotrophia adjacens, bifidobacterial and capnocytophaga. The oral_voting_transfer method employed three deep learning features, including TAGPPI, SeqVec and ProSE.

The performance of the models trained on the Streptococcus mutans dataset was poor for all three feature extraction methods. Among the three methods, ProSE showed the best stability and accuracy, making it the optimal choice. SeqVec, on the other hand, exhibited limitations and its model performance was generally less stable. However, it can be used for feature extraction in specific microbial protein data, as it excels in capturing positive sample labels with high coverage. TAGPPI performed comparably to ProSE, but considering the case of Streptococcus mutans, ProSE is the preferred choice. However, TAGPPI offers the advantage of being simpler and more efficient in terms of feature extraction and training. If large-scale services and training are required, TAGPPI would be the more favorable option.

## Data availability statement

The original contributions presented in the study are included in the article/Supplementary material, further inquiries can be directed to the corresponding author.

## Author contributions

WB: Data curation, Formal Analysis, Investigation, Resources, Software, Writing – original draft, Writing – review and editing. YL: Data curation, Formal Analysis, Resources, Writing – original draft, Writing – review and editing. BC: Conceptualization, Data curation, Investigation, Writing – review and editing, Writing – original draft.
