# Supplementary material for: Oral_voting_transfer: classification of oral microorganisms’ function proteins with voting transfer model
Source: Front Microbiol. 2024 Feb 7;14:1277121. doi: 10.3389/fmicb.2023.1277121 (PMC10879614; doi:10.3389/fmicb.2023.1277121)
Supplement: Supplementary file 1 [file Table_1.docx]

Table S1 The Comparison between Voting transfer learning model and art-of-the state classification model with TAGPPI, SeqVec, ProSE features in Streptococcus mutans's active sites

| Feature | Methods | ACC | Recall | F1 | Sn | Sp | MCC |
| --- | --- | --- | --- | --- | --- | --- | --- |
| TAGPPI | MLP | 73.76% | 0.0000 | 0.0000 | 100.00% | 0.00% | 0.0000 |
|  | Gaussian_Process | 68.09% | 0.7027 | 0.5361 | 67.30% | 70.30% | 0.3762 |
|  | Gaussian_NB | 70.92% | 0.7568 | 0.5773 | 69.20% | 75.70% | 0.4500 |
|  | Bernoulli_NB | 68.09% | 0.3243 | 0.3478 | 80.80% | 32.40% | 0.1508 |
|  | Decision Tree | 74.47% | 0.0541 | 0.1000 | 99.00% | 5.40% | 0.1250 |
|  | Bagging | 74.47% | 0.1081 | 0.1818 | 97.10% | 10.80% | 0.1564 |
|  | Random Forest | 68.79% | 0.2432 | 0.2903 | 84.60% | 24.30% | 0.1116 |
|  | AdaBoost | 70.21% | 0.3784 | 0.4000 | 81.70% | 37.80% | 0.2170 |
|  | Gradient Boosting | 70.21% | 0.1351 | 0.1923 | 90.40% | 13.50% | 0.0610 |
|  | Hist Gradient Boosting | 66.67% | 0.2432 | 0.2769 | 81.70% | 24.30% | 0.0733 |
|  | Voting Transfer Learning | 73.05% | 0.6000 | 0.6100 | 87.50% | 32.43% | 0.2388 |
| SeqVec | MLP | 73.76% | 0.0000 | 0.0000 | 100.00% | 0.00% | 0.0000 |
|  | Gaussian_Process | 47.52% | 0.5676 | 0.3621 | 44.20% | 56.80% | 0.0101 |
|  | Gaussian_NB | 73.76% | 0.0000 | 0.0000 | 100.00% | 0.00% | 0.0000 |
|  | Bernoulli_NB | 65.25% | 0.2703 | 0.2899 | 78.80% | 27.00% | 0.0678 |
|  | Decision Tree | 73.76% | 0.0000 | 0.0000 | 100.00% | 0.00% | 0.0000 |
|  | Bagging | 74.47% | 0.1081 | 0.1818 | 97.10% | 10.80% | 0.1564 |
|  | Random Forest | 65.25% | 0.1351 | 0.1695 | 83.70% | 13.50% | -0.0393 |
|  | AdaBoost | 61.70% | 0.0541 | 0.0690 | 81.70% | 5.40% | -0.1996 |
|  | Gradient Boosting | 70.21% | 0.0541 | 0.0870 | 93.30% | 5.40% | -0.0273 |
|  | Hist Gradient Boosting | 73.76% | 0.0000 | 0.0000 | 100.00% | 0.00% | 0.0000 |
|  | Voting Transfer Learning | 73.76% | 0.5100 | 0.4500 | 99.04% | 2.70% | 0.0649 |
| ProSE | MLP | 73.76% | 0.0000 | 0.0000 | 100.00% | 0.00% | 0.0000 |
|  | Gaussian_Process | 62.41% | 0.7027 | 0.4952 | 59.60% | 70.30% | 0.3007 |
|  | Gaussian_NB | 65.25% | 0.5946 | 0.4731 | 67.30% | 59.50% | 0.2688 |
|  | Bernoulli_NB | 64.54% | 0.3514 | 0.3421 | 75.00% | 35.10% | 0.1101 |
|  | Decision Tree | 73.05% | 0.1622 | 0.2400 | 93.30% | 16.20% | 0.1492 |
|  | Bagging | 71.63% | 0.1351 | 0.2000 | 92.30% | 13.50% | 0.0942 |
|  | Random Forest | 72.34% | 0.3243 | 0.3810 | 86.50% | 32.40% | 0.2247 |
|  | AdaBoost | 75.89% | 0.5405 | 0.5405 | 83.70% | 54.10% | 0.3957 |
|  | Gradient Boosting | 71.63% | 0.2432 | 0.3103 | 88.50% | 24.30% | 0.1669 |
|  | Hist Gradient Boosting | 70.92% | 0.2162 | 0.2807 | 88.50% | 21.60% | 0.1359 |
|  | Voting Transfer Learning | 73.05% | 0.5700 | 0.5800 | 90.38% | 24.32% | 0.1958 |

Table S2 The Comparison between Voting transfer learning model and art-of-the state classification model with TAGPPI, SeqVec, ProSE features in Streptococcus mutans's Binding sites

| Feature | Methods | ACC | Recall | F1 | Sn | Sp | MCC |
| --- | --- | --- | --- | --- | --- | --- | --- |
| TAGPPI | MLP | 74.47% | 0.7353 | 0.7353 | 75.30% | 73.50% | 0.4881 |
|  | Gaussian_Process | 75.18% | 0.8235 | 0.7619 | 68.50% | 82.40% | 0.5140 |
|  | Gaussian_NB | 76.60% | 0.8235 | 0.7724 | 71.20% | 82.40% | 0.5394 |
|  | Bernoulli_NB | 69.50% | 0.6912 | 0.6861 | 69.90% | 69.10% | 0.3900 |
|  | Decision Tree | 77.30% | 0.7647 | 0.7647 | 78.10% | 76.50% | 0.5461 |
|  | Bagging | 73.76% | 0.6765 | 0.7132 | 79.50% | 67.60% | 0.4744 |
|  | Random Forest | 77.30% | 0.7500 | 0.7612 | 79.50% | 75.00% | 0.5456 |
|  | AdaBoost | 75.89% | 0.7794 | 0.7571 | 74.00% | 77.90% | 0.5194 |
|  | Gradient Boosting | 74.47% | 0.7353 | 0.7353 | 75.30% | 73.50% | 0.4881 |
|  | Hist Gradient Boosting | 73.76% | 0.7500 | 0.7338 | 72.60% | 75.00% | 0.4761 |
|  | Voting Transfer Learning | 80.14% | 0.8000 | 0.8000 | 80.82% | 79.41% | 0.6024 |
| SeqVec | MLP | 56.03% | 0.4412 | 0.4918 | 67.10% | 44.10% | 0.1151 |
|  | Gaussian_Process | 53.90% | 0.6618 | 0.5806 | 42.50% | 66.20% | 0.0896 |
|  | Gaussian_NB | 56.03% | 0.4559 | 0.5000 | 65.80% | 45.60% | 0.1164 |
|  | Bernoulli_NB | 47.52% | 0.4559 | 0.4559 | 49.30% | 45.60% | -0.0510 |
|  | Decision Tree | 51.77% | 0.0000 | 0.0000 | 100.00% | 0.00% | 0.0000 |
|  | Bagging | 55.32% | 0.4118 | 0.4706 | 68.50% | 41.20% | 0.1008 |
|  | Random Forest | 57.45% | 0.5147 | 0.5385 | 63.00% | 51.50% | 0.1460 |
|  | AdaBoost | 53.90% | 0.5294 | 0.5255 | 54.80% | 52.90% | 0.0770 |
|  | Gradient Boosting | 56.03% | 0.5441 | 0.5441 | 57.50% | 54.40% | 0.1191 |
|  | Hist Gradient Boosting | 54.61% | 0.5147 | 0.5224 | 57.50% | 51.50% | 0.0902 |
|  | Voting Transfer Learning | 56.03% | 0.5600 | 0.5500 | 68.49% | 42.65% | 0.1153 |
| ProSE | MLP | 52.48% | 0.0147 | 0.0290 | 100.00% | 1.50% | 0.0869 |
|  | Gaussian_Process | 57.45% | 0.9412 | 0.6809 | 23.30% | 94.10% | 0.2464 |
|  | Gaussian_NB | 81.56% | 0.9265 | 0.8289 | 71.20% | 92.60% | 0.6531 |
|  | Bernoulli_NB | 69.50% | 0.6912 | 0.6861 | 69.90% | 69.10% | 0.3900 |
|  | Decision Tree | 80.85% | 0.8088 | 0.8029 | 80.80% | 80.90% | 0.6170 |
|  | Bagging | 74.47% | 0.6912 | 0.7231 | 79.50% | 69.10% | 0.4886 |
|  | Random Forest | 82.27% | 0.8382 | 0.8201 | 80.80% | 83.80% | 0.6463 |
|  | AdaBoost | 75.18% | 0.7500 | 0.7445 | 75.30% | 75.00% | 0.5030 |
|  | Gradient Boosting | 78.72% | 0.7500 | 0.7727 | 82.20% | 75.00% | 0.5735 |
|  | Hist Gradient Boosting | 74.47% | 0.6324 | 0.7049 | 84.90% | 63.20% | 0.4927 |
|  | Voting Transfer Learning | 75.18% | 0.7500 | 0.7500 | 71.23% | 79.41% | 0.5081 |

Table S3 The Comparison between Voting transfer learning model and art-of-the state classification model with TAGPPI, SeqVec, ProSE features in Staphylococcus aureus's active sites

| Feature | Methods | ACC | Recall | F1 | Sn | Sp | MCC |
| --- | --- | --- | --- | --- | --- | --- | --- |
| TAGPPI | MLP | 96.55% | 0.8627 | 0.8889 | 98.50% | 86.30% | 0.8544 |
|  | Gaussian_Process | 73.04% | 0.8039 | 0.4881 | 71.60% | 80.40% | 0.5220 |
|  | Gaussian_NB | 75.55% | 0.8235 | 0.5185 | 74.30% | 82.40% | 0.5689 |
|  | Bernoulli_NB | 90.91% | 0.7059 | 0.7129 | 94.80% | 70.60% | 0.6740 |
|  | Decision Tree | 89.03% | 0.3137 | 0.4776 | 100.00% | 31.40% | 0.4316 |
|  | Bagging | 94.98% | 0.7255 | 0.8222 | 99.30% | 72.50% | 0.7453 |
|  | Random Forest | 93.10% | 0.7059 | 0.7660 | 97.40% | 70.60% | 0.7058 |
|  | AdaBoost | 91.22% | 0.6667 | 0.7083 | 95.90% | 66.70% | 0.6545 |
|  | Gradient Boosting | 95.92% | 0.7451 | 0.8539 | 100.00% | 74.50% | 0.7705 |
|  | Hist Gradient Boosting | 96.24% | 0.8627 | 0.8800 | 98.10% | 86.30% | 0.8499 |
|  | Voting Transfer Learning | 94.98% | 0.8700 | 0.9000 | 98.51% | 76.47% | 0.7687 |
| SeqVec | MLP | 91.85% | 0.5686 | 0.6905 | 98.50% | 56.90% | 0.6092 |
|  | Gaussian_Process | 60.19% | 0.7059 | 0.3618 | 58.20% | 70.60% | 0.2902 |
|  | Gaussian_NB | 84.33% | 0.0196 | 0.0385 | 100.00% | 2.00% | 0.1005 |
|  | Bernoulli_NB | 84.64% | 0.4902 | 0.5051 | 91.40% | 49.00% | 0.4461 |
|  | Decision Tree | 84.01% | 0.0000 | 0.0000 | 100.00% | 0.00% | 0.0000 |
|  | Bagging | 89.66% | 0.3725 | 0.5352 | 99.60% | 37.30% | 0.4717 |
|  | Random Forest | 88.71% | 0.4118 | 0.5385 | 97.80% | 41.20% | 0.4731 |
|  | AdaBoost | 89.34% | 0.4902 | 0.5952 | 97.00% | 49.00% | 0.5244 |
|  | Gradient Boosting | 90.28% | 0.3922 | 0.5634 | 100.00% | 39.20% | 0.4937 |
|  | Hist Gradient Boosting | 84.01% | 0.0000 | 0.0000 | 100.00% | 0.00% | 0.0000 |
|  | Voting Transfer Learning | 88.71% | 0.6500 | 0.7000 | 100.00% | 29.41% | 0.4152 |
| ProSE | MLP | 88.09% | 0.2549 | 0.4063 | 100.00% | 25.50% | 0.3823 |
|  | Gaussian_Process | 81.19% | 0.5294 | 0.4737 | 86.60% | 52.90% | 0.4195 |
|  | Gaussian_NB | 77.12% | 0.8235 | 0.5350 | 76.10% | 82.40% | 0.5862 |
|  | Bernoulli_NB | 94.04% | 0.8235 | 0.8155 | 96.30% | 82.40% | 0.7947 |
|  | Decision Tree | 93.10% | 0.6667 | 0.7556 | 98.10% | 66.70% | 0.6825 |
|  | Bagging | 94.98% | 0.6863 | 0.8140 | 100.00% | 68.60% | 0.7225 |
|  | Random Forest | 97.49% | 0.8627 | 0.9167 | 99.60% | 86.30% | 0.8667 |
|  | AdaBoost | 94.98% | 0.7647 | 0.8298 | 98.50% | 76.50% | 0.7688 |
|  | Gradient Boosting | 97.18% | 0.8627 | 0.9072 | 99.30% | 86.30% | 0.8633 |
|  | Hist Gradient Boosting | 94.36% | 0.7843 | 0.8163 | 97.40% | 78.40% | 0.7721 |
|  | Voting Transfer Learning | 95.30% | 0.8700 | 0.9000 | 99.25% | 74.51% | 0.7613 |

Table S4 The Comparison between Voting transfer learning model and art-of-the state classification model with TAGPPI, SeqVec, ProSE features in Staphylococcus aureus's binding sites

| Feature | Methods | ACC | Recall | F1 | Sn | Sp | MCC |
| --- | --- | --- | --- | --- | --- | --- | --- |
| TAGPPI | MLP | 95.61% | 0.9586 | 0.9521 | 95.40% | 95.90% | 0.9130 |
|  | Gaussian_Process | 78.68% | 0.8966 | 0.7927 | 69.50% | 89.70% | 0.6045 |
|  | Gaussian_NB | 79.31% | 0.8897 | 0.7963 | 71.30% | 89.00% | 0.6127 |
|  | Bernoulli_NB | 88.09% | 0.9103 | 0.8742 | 85.60% | 91.00% | 0.7671 |
|  | Decision Tree | 86.83% | 0.9034 | 0.8618 | 83.90% | 90.30% | 0.7435 |
|  | Bagging | 89.34% | 0.8621 | 0.8803 | 92.00% | 86.20% | 0.7833 |
|  | Random Forest | 92.48% | 0.9103 | 0.9167 | 93.70% | 91.00% | 0.8473 |
|  | AdaBoost | 89.97% | 0.9103 | 0.8919 | 89.10% | 91.00% | 0.8011 |
|  | Gradient Boosting | 95.61% | 0.9586 | 0.9521 | 95.40% | 95.90% | 0.9130 |
|  | Hist Gradient Boosting | 96.55% | 0.9586 | 0.9619 | 97.10% | 95.90% | 0.9301 |
|  | Voting Transfer Learning | 95.61% | 0.9600 | 0.9600 | 95.98% | 95.17% | 0.9115 |
| SeqVec | MLP | 89.97% | 0.8759 | 0.8881 | 92.00% | 87.60% | 0.7968 |
|  | Gaussian_Process | 61.44% | 0.7655 | 0.6435 | 48.90% | 76.60% | 0.2654 |
|  | Gaussian_NB | 63.64% | 0.6483 | 0.6184 | 62.60% | 64.80% | 0.2741 |
|  | Bernoulli_NB | 74.61% | 0.7310 | 0.7235 | 75.90% | 73.10% | 0.4902 |
|  | Decision Tree | 54.55% | 0.0000 | 0.0000 | 100.00% | 0.00% | 0.0000 |
|  | Bagging | 83.70% | 0.7655 | 0.8102 | 89.70% | 76.60% | 0.6688 |
|  | Random Forest | 79.31% | 0.7172 | 0.7591 | 85.60% | 71.70% | 0.5786 |
|  | AdaBoost | 82.13% | 0.7517 | 0.7927 | 87.90% | 75.20% | 0.6362 |
|  | Gradient Boosting | 81.82% | 0.7448 | 0.7883 | 87.90% | 74.50% | 0.6297 |
|  | Hist Gradient Boosting | 63.01% | 0.8276 | 0.6704 | 46.60% | 82.80% | 0.3154 |
|  | Voting Transfer Learning | 82.76% | 0.8200 | 0.8300 | 86.78% | 77.93% | 0.6496 |
| ProSE | MLP | 64.89% | 0.2276 | 0.3708 | 100.00% | 22.80% | 0.3587 |
|  | Gaussian_Process | 69.28% | 0.4897 | 0.5917 | 86.20% | 49.00% | 0.3792 |
|  | Gaussian_NB | 79.94% | 0.8414 | 0.7922 | 76.40% | 84.10% | 0.6068 |
|  | Bernoulli_NB | 91.85% | 0.8897 | 0.9085 | 94.30% | 89.00% | 0.8342 |
|  | Decision Tree | 86.21% | 0.8276 | 0.8451 | 89.10% | 82.80% | 0.7204 |
|  | Bagging | 93.73% | 0.8966 | 0.9286 | 97.10% | 89.70% | 0.8704 |
|  | Random Forest | 95.61% | 0.9448 | 0.9514 | 96.60% | 94.50% | 0.9112 |
|  | AdaBoost | 92.79% | 0.8966 | 0.9187 | 95.40% | 89.70% | 0.8524 |
|  | Gradient Boosting | 96.87% | 0.9586 | 0.9653 | 97.70% | 95.90% | 0.9362 |
|  | Hist Gradient Boosting | 95.61% | 0.9448 | 0.9514 | 96.60% | 94.50% | 0.9112 |
|  | Voting Transfer Learning | 93.42% | 0.9400 | 0.9300 | 91.38% | 95.86% | 0.8733 |

Table S5 The Comparison between Voting transfer learning model and art-of-the state classification model with TAGPPI, SeqVec, ProSE features in Abiotrophia adjacent active sites

| Feature | Methods | ACC | Recall | F1 | Sn | Sp | MCC |
| --- | --- | --- | --- | --- | --- | --- | --- |
| TAGPPI | MLP | 95.54% | 0.7518 | 0.8061 | 98.40% | 75.20% | 0.7566 |
|  | Gaussian_Process | 75.68% | 0.8582 | 0.4654 | 74.30% | 85.80% | 0.6050 |
|  | Gaussian_NB | 76.99% | 0.7872 | 0.4577 | 76.70% | 78.70% | 0.5541 |
|  | Bernoulli_NB | 89.85% | 0.6170 | 0.6000 | 93.80% | 61.70% | 0.5860 |
|  | Decision Tree | 89.68% | 0.2837 | 0.4040 | 98.30% | 28.40% | 0.3734 |
|  | Bagging | 92.65% | 0.4539 | 0.6038 | 99.30% | 45.40% | 0.5307 |
|  | Random Forest | 93.18% | 0.6383 | 0.6977 | 97.30% | 63.80% | 0.6485 |
|  | AdaBoost | 90.20% | 0.4468 | 0.5294 | 96.60% | 44.70% | 0.4832 |
|  | Gradient Boosting | 95.28% | 0.6525 | 0.7731 | 99.50% | 65.20% | 0.6888 |
|  | Hist Gradient Boosting | 95.63% | 0.7730 | 0.8134 | 98.20% | 77.30% | 0.7721 |
|  | Voting Transfer Learning | 94.98% | 0.8700 | 0.9000 | 98.51% | 76.47% | 0.7687 |
| SeqVec | MLP | 93.26% | 0.5106 | 0.6516 | 99.20% | 51.10% | 0.5737 |
|  | Gaussian_Process | 55.03% | 0.6667 | 0.2678 | 53.40% | 66.70% | 0.2028 |
|  | Gaussian_NB | 87.05% | 0.0071 | 0.0133 | 99.20% | 0.70% | -0.0058 |
|  | Bernoulli_NB | 87.05% | 0.4965 | 0.4861 | 92.30% | 49.60% | 0.4634 |
|  | Decision Tree | 87.66% | 0.0000 | 0.0000 | 100.00% | 0.00% | 0.0000 |
|  | Bagging | 90.73% | 0.2766 | 0.4239 | 99.60% | 27.70% | 0.3928 |
|  | Random Forest | 87.58% | 0.2340 | 0.3173 | 96.60% | 23.40% | 0.2936 |
|  | AdaBoost | 87.66% | 0.0213 | 0.0408 | 99.70% | 2.10% | 0.0827 |
|  | Gradient Boosting | 92.48% | 0.3972 | 0.5657 | 99.90% | 39.70% | 0.4959 |
|  | Hist Gradient Boosting | 87.66% | 0.0000 | 0.0000 | 100.00% | 0.00% | 0.0000 |
|  | Voting Transfer Learning | 88.71% | 0.6500 | 0.7000 | 100.00% | 29.41% | 0.4152 |
| ProSE | MLP | 90.81% | 0.2553 | 0.4068 | 100.00% | 25.50% | 0.3823 |
|  | Gaussian_Process | 78.92% | 0.7660 | 0.4726 | 79.20% | 76.60% | 0.5582 |
|  | Gaussian_NB | 76.47% | 0.8794 | 0.4797 | 74.90% | 87.90% | 0.6334 |
|  | Bernoulli_NB | 94.14% | 0.7376 | 0.7564 | 97.00% | 73.80% | 0.7279 |
|  | Decision Tree | 92.04% | 0.5248 | 0.6192 | 97.60% | 52.50% | 0.5613 |
|  | Bagging | 94.23% | 0.6383 | 0.7317 | 98.50% | 63.80% | 0.6643 |
|  | Random Forest | 92.65% | 0.6596 | 0.6889 | 96.40% | 66.00% | 0.6550 |
|  | AdaBoost | 92.30% | 0.7021 | 0.6923 | 95.40% | 70.20% | 0.6779 |
|  | Gradient Boosting | 96.24% | 0.8085 | 0.8413 | 98.40% | 80.90% | 0.8054 |
|  | Hist Gradient Boosting | 93.61% | 0.6312 | 0.7092 | 97.90% | 63.10% | 0.6507 |
|  | Voting Transfer Learning | 95.30% | 0.8700 | 0.9000 | 99.25% | 74.51% | 0.7613 |

Table S6 The Comparison between Voting transfer learning model and art-of-the state classification model with TAGPPI, SeqVec, ProSE features in Abiotrophia adjacent binding sites

| Feature | Methods | ACC | Recall | F1 | Sn | Sp | MCC |
| --- | --- | --- | --- | --- | --- | --- | --- |
| TAGPPI | MLP | 95.54% | 0.7518 | 0.8061 | 98.40% | 75.20% | 0.7566 |
|  | Gaussian_Process | 75.68% | 0.8582 | 0.4654 | 74.30% | 85.80% | 0.6050 |
|  | Gaussian_NB | 76.99% | 0.7872 | 0.4577 | 76.70% | 78.70% | 0.5541 |
|  | Bernoulli_NB | 90.64% | 0.6879 | 0.6445 | 93.70% | 68.80% | 0.6453 |
|  | Decision Tree | 89.76% | 0.2766 | 0.4000 | 98.50% | 27.70% | 0.3710 |
|  | Bagging | 92.21% | 0.4326 | 0.5782 | 99.10% | 43.30% | 0.5109 |
|  | Random Forest | 93.18% | 0.6383 | 0.6977 | 97.30% | 63.80% | 0.6485 |
|  | AdaBoost | 90.20% | 0.4468 | 0.5294 | 96.60% | 44.70% | 0.4832 |
|  | Gradient Boosting | 95.28% | 0.6525 | 0.7731 | 99.50% | 65.20% | 0.6888 |
|  | Hist Gradient Boosting | 95.63% | 0.7730 | 0.8134 | 98.20% | 77.30% | 0.7721 |
|  | Voting Transfer Learning | 95.61% | 0.9600 | 0.9600 | 95.98% | 95.17% | 0.9115 |
| SeqVec | MLP | 93.26% | 0.5106 | 0.6516 | 99.20% | 51.10% | 0.5737 |
|  | Gaussian_Process | 55.03% | 0.6667 | 0.2678 | 53.40% | 66.70% | 0.2028 |
|  | Gaussian_NB | 87.05% | 0.0071 | 0.0133 | 99.20% | 0.70% | -0.0058 |
|  | Bernoulli_NB | 87.84% | 0.4965 | 0.5018 | 93.20% | 49.60% | 0.4756 |
|  | Decision Tree | 87.66% | 0.0000 | 0.0000 | 100.00% | 0.00% | 0.0000 |
|  | Bagging | 89.85% | 0.2199 | 0.3483 | 99.40% | 22.00% | 0.3380 |
|  | Random Forest | 87.58% | 0.2340 | 0.3173 | 96.60% | 23.40% | 0.2936 |
|  | AdaBoost | 87.66% | 0.0213 | 0.0408 | 99.70% | 2.10% | 0.0827 |
|  | Gradient Boosting | 92.48% | 0.3972 | 0.5657 | 99.90% | 39.70% | 0.4959 |
|  | Hist Gradient Boosting | 87.66% | 0.0000 | 0.0000 | 100.00% | 0.00% | 0.0000 |
|  | Voting Transfer Learning | 82.76% | 0.8200 | 0.8300 | 86.78% | 77.93% | 0.6496 |
| ProSE | MLP | 90.81% | 0.2553 | 0.4068 | 100.00% | 25.50% | 0.3823 |
|  | Gaussian_Process | 78.92% | 0.7660 | 0.4726 | 79.20% | 76.60% | 0.5582 |
|  | Gaussian_NB | 76.47% | 0.8794 | 0.4797 | 74.90% | 87.90% | 0.6334 |
|  | Bernoulli_NB | 93.26% | 0.7518 | 0.7336 | 95.80% | 75.20% | 0.7256 |
|  | Decision Tree | 92.21% | 0.5177 | 0.6213 | 97.90% | 51.80% | 0.5601 |
|  | Bagging | 94.14% | 0.6383 | 0.7287 | 98.40% | 63.80% | 0.6629 |
|  | Random Forest | 92.65% | 0.6596 | 0.6889 | 96.40% | 66.00% | 0.6550 |
|  | AdaBoost | 92.30% | 0.7021 | 0.6923 | 95.40% | 70.20% | 0.6779 |
|  | Gradient Boosting | 96.24% | 0.8085 | 0.8413 | 98.40% | 80.90% | 0.8054 |
|  | Hist Gradient Boosting | 93.61% | 0.6312 | 0.7092 | 97.90% | 63.10% | 0.6507 |
|  | Voting Transfer Learning | 93.42% | 0.9400 | 0.9300 | 91.38% | 95.86% | 0.8733 |

Table S7 The Comparison between Voting transfer learning model and art-of-the state classification model with TAGPPI, SeqVec, ProSE features in Bifidobacterial's active sites

| Feature | Methods | ACC | Recall | F1 | Sn | Sp | MCC |
| --- | --- | --- | --- | --- | --- | --- | --- |
| TAGPPI | MLP | 96.55% | 0.8627 | 0.8889 | 98.50% | 86.30% | 0.8544 |
|  | Gaussian_Process | 73.04% | 0.8039 | 0.4881 | 71.60% | 80.40% | 0.5220 |
|  | Gaussian_NB | 75.55% | 0.8235 | 0.5185 | 74.30% | 82.40% | 0.5689 |
|  | Bernoulli_NB | 91.22% | 0.7451 | 0.7308 | 94.40% | 74.50% | 0.7031 |
|  | Decision Tree | 89.03% | 0.3725 | 0.5205 | 98.90% | 37.30% | 0.4595 |
|  | Bagging | 93.42% | 0.6275 | 0.7529 | 99.30% | 62.70% | 0.6662 |
|  | Random Forest | 93.10% | 0.7059 | 0.7660 | 97.40% | 70.60% | 0.7058 |
|  | AdaBoost | 91.22% | 0.6667 | 0.7083 | 95.90% | 66.70% | 0.6545 |
|  | Gradient Boosting | 95.92% | 0.7451 | 0.8539 | 100.00% | 74.50% | 0.7705 |
|  | Hist Gradient Boosting | 96.24% | 0.8627 | 0.8800 | 98.10% | 86.30% | 0.8499 |
|  | Voting Transfer Learning | 95.30% | 0.8800 | 0.9100 | 98.88% | 76.47% | 0.7732 |
| SeqVec | MLP | 91.85% | 0.5686 | 0.6905 | 98.50% | 56.90% | 0.6092 |
|  | Gaussian_Process | 60.19% | 0.7059 | 0.3618 | 58.20% | 70.60% | 0.2902 |
|  | Gaussian_NB | 84.33% | 0.0196 | 0.0385 | 100.00% | 2.00% | 0.1005 |
|  | Bernoulli_NB | 83.70% | 0.4314 | 0.4583 | 91.40% | 43.10% | 0.3940 |
|  | Decision Tree | 84.01% | 0.0000 | 0.0000 | 100.00% | 0.00% | 0.0000 |
|  | Bagging | 89.34% | 0.3529 | 0.5143 | 99.60% | 35.30% | 0.4557 |
|  | Random Forest | 88.71% | 0.4118 | 0.5385 | 97.80% | 41.20% | 0.4731 |
|  | AdaBoost | 89.34% | 0.4902 | 0.5952 | 97.00% | 49.00% | 0.5244 |
|  | Gradient Boosting | 90.28% | 0.3922 | 0.5634 | 100.00% | 39.20% | 0.4937 |
|  | Hist Gradient Boosting | 84.01% | 0.0000 | 0.0000 | 100.00% | 0.00% | 0.0000 |
|  | Voting Transfer Learning | 89.34% | 0.6700 | 0.7200 | 100.00% | 33.33% | 0.4472 |
| ProSE | MLP | 88.09% | 0.2549 | 0.4063 | 100.00% | 25.50% | 0.3823 |
|  | Gaussian_Process | 81.19% | 0.5294 | 0.4737 | 86.60% | 52.90% | 0.4195 |
|  | Gaussian_NB | 77.12% | 0.8235 | 0.5350 | 76.10% | 82.40% | 0.5862 |
|  | Bernoulli_NB | 92.48% | 0.7451 | 0.7600 | 95.90% | 74.50% | 0.7207 |
|  | Decision Tree | 92.48% | 0.6078 | 0.7209 | 98.50% | 60.80% | 0.6402 |
|  | Bagging | 95.61% | 0.7255 | 0.8409 | 100.00% | 72.50% | 0.7541 |
|  | Random Forest | 97.49% | 0.8627 | 0.9167 | 99.60% | 86.30% | 0.8667 |
|  | AdaBoost | 94.98% | 0.7647 | 0.8298 | 98.50% | 76.50% | 0.7688 |
|  | Gradient Boosting | 97.18% | 0.8627 | 0.9072 | 99.30% | 86.30% | 0.8633 |
|  | Hist Gradient Boosting | 94.36% | 0.7843 | 0.8163 | 97.40% | 78.40% | 0.7721 |
|  | Voting Transfer Learning | 95.30% | 0.8700 | 0.9000 | 99.25% | 74.51% | 0.7613 |

Table S8 The Comparison between Voting transfer learning model and art-of-the state classification model with TAGPPI, SeqVec, ProSE features in Bifidobacterial's binding sites

| Feature | Methods | ACC | Recall | F1 | Sn | Sp | MCC |
| --- | --- | --- | --- | --- | --- | --- | --- |
| TAGPPI | MLP | 95.61% | 0.9586 | 0.9521 | 95.40% | 95.90% | 0.9130 |
|  | Gaussian_Process | 78.68% | 0.8966 | 0.7927 | 69.50% | 89.70% | 0.6045 |
|  | Gaussian_NB | 79.31% | 0.8897 | 0.7963 | 71.30% | 89.00% | 0.6127 |
|  | Bernoulli_NB | 86.83% | 0.8759 | 0.8581 | 86.20% | 87.60% | 0.7381 |
|  | Decision Tree | 87.77% | 0.9310 | 0.8738 | 83.30% | 93.10% | 0.7677 |
|  | Bagging | 88.71% | 0.8621 | 0.8741 | 90.80% | 86.20% | 0.7708 |
|  | Random Forest | 92.48% | 0.9103 | 0.9167 | 93.70% | 91.00% | 0.8473 |
|  | AdaBoost | 89.97% | 0.9103 | 0.8919 | 89.10% | 91.00% | 0.8011 |
|  | Gradient Boosting | 95.61% | 0.9586 | 0.9521 | 95.40% | 95.90% | 0.9130 |
|  | Hist Gradient Boosting | 96.55% | 0.9586 | 0.9619 | 97.10% | 95.90% | 0.9301 |
|  | Voting Transfer Learning | 94.67% | 0.95 | 0.95 | 95.98% | 93.10% | 0.8912 |
| SeqVec | MLP | 89.97% | 0.8759 | 0.8881 | 92.00% | 87.60% | 0.7968 |
|  | Gaussian_Process | 61.44% | 0.7655 | 0.6435 | 48.90% | 76.60% | 0.2654 |
|  | Gaussian_NB | 63.64% | 0.6483 | 0.6184 | 62.60% | 64.80% | 0.2741 |
|  | Bernoulli_NB | 75.55% | 0.7655 | 0.7400 | 74.70% | 76.60% | 0.5131 |
|  | Decision Tree | 54.55% | 0.0000 | 0.0000 | 100.00% | 0.00% | 0.0000 |
|  | Bagging | 77.43% | 0.6759 | 0.7313 | 85.60% | 67.60% | 0.5408 |
|  | Random Forest | 79.31% | 0.7172 | 0.7591 | 85.60% | 71.70% | 0.5786 |
|  | AdaBoost | 82.13% | 0.7517 | 0.7927 | 87.90% | 75.20% | 0.6362 |
|  | Gradient Boosting | 81.82% | 0.7448 | 0.7883 | 87.90% | 74.50% | 0.6297 |
|  | Hist Gradient Boosting | 63.01% | 0.8276 | 0.6704 | 46.60% | 82.80% | 0.3154 |
|  | Voting Transfer Learning | 81.19% | 0.8000 | 0.8100 | 89.08% | 71.72% | 0.6174 |
| ProSE | MLP | 64.89% | 0.2276 | 0.3708 | 100.00% | 22.80% | 0.3587 |
|  | Gaussian_Process | 69.28% | 0.4897 | 0.5917 | 86.20% | 49.00% | 0.3792 |
|  | Gaussian_NB | 79.94% | 0.8414 | 0.7922 | 76.40% | 84.10% | 0.6068 |
|  | Bernoulli_NB | 92.79% | 0.9103 | 0.9199 | 94.30% | 91.00% | 0.8535 |
|  | Decision Tree | 86.52% | 0.8345 | 0.8491 | 89.10% | 83.40% | 0.7262 |
|  | Bagging | 93.10% | 0.8759 | 0.9203 | 97.70% | 87.60% | 0.8574 |
|  | Random Forest | 95.61% | 0.9448 | 0.9514 | 96.60% | 94.50% | 0.9112 |
|  | AdaBoost | 92.79% | 0.8966 | 0.9187 | 95.40% | 89.70% | 0.8524 |
|  | Gradient Boosting | 96.87% | 0.9586 | 0.9653 | 97.70% | 95.90% | 0.9362 |
|  | Hist Gradient Boosting | 95.61% | 0.9448 | 0.9514 | 96.60% | 94.50% | 0.9112 |
|  | Voting Transfer Learning | 93.42% | 0.9400 | 0.9300 | 91.95% | 95.17% | 0.8717 |

Table S9 The Comparison between Voting transfer learning model and art-of-the state classification model with TAGPPI, SeqVec, ProSE features in Capnocytophaga's active sites

| Feature | Methods | ACC | Recall | F1 | Sn | Sp | MCC |
| --- | --- | --- | --- | --- | --- | --- | --- |
| TAGPPI | MLP | 97.94% | 0.6627 | 0.7746 | 99.70% | 66.30% | 0.7002 |
|  | Gaussian_Process | 81.68% | 0.7831 | 0.3140 | 81.90% | 78.30% | 0.6024 |
|  | Gaussian_NB | 83.10% | 0.7229 | 0.3141 | 83.70% | 72.30% | 0.5637 |
|  | Bernoulli_NB | 95.55% | 0.6145 | 0.5965 | 97.50% | 61.40% | 0.6316 |
|  | Decision Tree | 94.65% | 0.0000 | 0.0000 | 100.00% | 0.00% | 0.0000 |
|  | Bagging | 97.23% | 0.4940 | 0.6560 | 99.90% | 49.40% | 0.5712 |
|  | Random Forest | 95.87% | 0.4819 | 0.5556 | 98.60% | 48.20% | 0.5419 |
|  | AdaBoost | 94.65% | 0.3614 | 0.4196 | 98.00% | 36.10% | 0.4342 |
|  | Gradient Boosting | 97.87% | 0.6265 | 0.7591 | 99.90% | 62.70% | 0.6744 |
|  | Hist Gradient Boosting | 97.48% | 0.6747 | 0.7417 | 99.20% | 67.50% | 0.7033 |
|  | Voting Transfer Learning | 97.68% | 0.8600 | 0.8800 | 99.05% | 73.49% | 0.7503 |
| SeqVec | MLP | 94.65% | 0.0000 | 0.0000 | 100.00% | 0.00% | 0.0000 |
|  | Gaussian_Process | 56.06% | 0.5904 | 0.1258 | 55.90% | 59.00% | 0.1491 |
|  | Gaussian_NB | 94.52% | 0.0000 | 0.0000 | 99.90% | 0.00% | -0.0224 |
|  | Bernoulli_NB | 93.29% | 0.5542 | 0.4694 | 95.40% | 55.40% | 0.5543 |
|  | Decision Tree | 94.65% | 0.0000 | 0.0000 | 100.00% | 0.00% | 0.0000 |
|  | Bagging | 95.87% | 0.2530 | 0.3962 | 99.90% | 25.30% | 0.3784 |
|  | Random Forest | 95.03% | 0.2289 | 0.3304 | 99.10% | 22.90% | 0.3397 |
|  | AdaBoost | 93.74% | 0.0241 | 0.0396 | 98.90% | 2.40% | 0.0496 |
|  | Gradient Boosting | 97.03% | 0.4578 | 0.6230 | 99.90% | 45.80% | 0.5434 |
|  | Hist Gradient Boosting | 94.65% | 0.0000 | 0.0000 | 100.00% | 0.00% | 0.0000 |
|  | Voting Transfer Learning | 94.65% | 0.5000 | 0.4900 | 100.00% | 0.00% | 0.0000 |
| ProSE | MLP | 96.77% | 0.3976 | 0.5690 | 100.00% | 39.80% | 0.4984 |
|  | Gaussian_Process | 39.55% | 0.9277 | 0.1412 | 36.50% | 92.80% | 0.3545 |
|  | Gaussian_NB | 80.32% | 0.8675 | 0.3207 | 80.00% | 86.70% | 0.6685 |
|  | Bernoulli_NB | 96.06% | 0.6988 | 0.6554 | 97.50% | 69.90% | 0.7012 |
|  | Decision Tree | 95.10% | 0.1446 | 0.2400 | 99.70% | 14.50% | 0.2712 |
|  | Bagging | 97.03% | 0.4940 | 0.6406 | 99.70% | 49.40% | 0.5681 |
|  | Random Forest | 97.55% | 0.6506 | 0.7397 | 99.40% | 65.10% | 0.6867 |
|  | AdaBoost | 95.23% | 0.3735 | 0.4559 | 98.50% | 37.30% | 0.4527 |
|  | Gradient Boosting | 98.00% | 0.6506 | 0.7770 | 99.90% | 65.10% | 0.6933 |
|  | Hist Gradient Boosting | 98.00% | 0.7711 | 0.8050 | 99.20% | 77.10% | 0.7823 |
|  | Voting Transfer Learning | 97.35% | 0.7800 | 0.8400 | 99.66% | 56.63% | 0.6236 |

Table S10 The Comparison between Voting transfer learning model and art-of-the state classification model with TAGPPI, SeqVec, ProSE features in Capnocytophaga's binding sites

| Feature | Methods | ACC | Recall | F1 | Sn | Sp | MCC |
| --- | --- | --- | --- | --- | --- | --- | --- |
| TAGPPI | MLP | 97.29% | 0.7744 | 0.8306 | 99.20% | 77.40% | 0.7849 |
|  | Gaussian_Process | 80.77% | 0.8421 | 0.4291 | 80.50% | 84.20% | 0.6474 |
|  | Gaussian_NB | 81.55% | 0.8271 | 0.4348 | 81.40% | 82.70% | 0.6411 |
|  | Bernoulli_NB | 93.35% | 0.6466 | 0.6255 | 96.00% | 64.70% | 0.6391 |
|  | Decision Tree | 92.90% | 0.1880 | 0.3125 | 99.90% | 18.80% | 0.3196 |
|  | Bagging | 95.42% | 0.4962 | 0.6502 | 99.70% | 49.60% | 0.5696 |
|  | Random Forest | 93.94% | 0.5338 | 0.6017 | 97.70% | 53.40% | 0.5700 |
|  | AdaBoost | 93.23% | 0.5338 | 0.5749 | 97.00% | 53.40% | 0.5600 |
|  | Gradient Boosting | 97.48% | 0.7218 | 0.8312 | 99.90% | 72.20% | 0.7504 |
|  | Hist Gradient Boosting | 97.42% | 0.7970 | 0.8413 | 99.10% | 79.70% | 0.8033 |
|  | Voting Transfer Learning | 97.55% | 0.8800 | 0.9200 | 99.44% | 77.44% | 0.7881 |
| SeqVec | MLP | 95.74% | 0.5639 | 0.6944 | 99.40% | 56.40% | 0.6181 |
|  | Gaussian_Process | 59.23% | 0.6466 | 0.2139 | 58.70% | 64.70% | 0.2344 |
|  | Gaussian_NB | 90.52% | 0.0451 | 0.0755 | 98.60% | 4.50% | 0.0916 |
|  | Bernoulli_NB | 90.71% | 0.5865 | 0.5200 | 93.70% | 58.60% | 0.5585 |
|  | Decision Tree | 91.42% | 0.0000 | 0.0000 | 100.00% | 0.00% | 0.0000 |
|  | Bagging | 93.42% | 0.2782 | 0.4205 | 99.60% | 27.80% | 0.3937 |
|  | Random Forest | 90.97% | 0.1654 | 0.2391 | 98.00% | 16.50% | 0.2502 |
|  | AdaBoost | 90.45% | 0.0752 | 0.1190 | 98.20% | 7.50% | 0.1353 |
|  | Gradient Boosting | 95.61% | 0.5038 | 0.6634 | 99.90% | 50.40% | 0.5789 |
|  | Hist Gradient Boosting | 91.42% | 0.0000 | 0.0000 | 100.00% | 0.00% | 0.0000 |
|  | Voting Transfer Learning | 93.48% | 0.6200 | 0.6800 | 99.93% | 24.81% | 0.3748 |
| ProSE | MLP | 94.90% | 0.4060 | 0.5775 | 100.00% | 40.60% | 0.5047 |
|  | Gaussian_Process | 77.29% | 0.8496 | 0.3910 | 76.60% | 85.00% | 0.6182 |
|  | Gaussian_NB | 80.13% | 0.8722 | 0.4296 | 79.50% | 87.20% | 0.6690 |
|  | Bernoulli_NB | 95.55% | 0.7444 | 0.7416 | 97.50% | 74.40% | 0.7390 |
|  | Decision Tree | 94.71% | 0.4211 | 0.5773 | 99.60% | 42.10% | 0.5097 |
|  | Bagging | 96.39% | 0.6391 | 0.7522 | 99.40% | 63.90% | 0.6771 |
|  | Random Forest | 96.26% | 0.7368 | 0.7717 | 98.40% | 73.70% | 0.7441 |
|  | AdaBoost | 96.39% | 0.7444 | 0.7795 | 98.40% | 74.40% | 0.7499 |
|  | Gradient Boosting | 97.87% | 0.7744 | 0.8619 | 99.80% | 77.40% | 0.7921 |
|  | Hist Gradient Boosting | 96.19% | 0.7970 | 0.7823 | 97.70% | 79.70% | 0.7869 |
|  | Voting Transfer Learning | 97.03% | 0.8500 | 0.8900 | 99.58% | 69.92% | 0.7277 |
